# Neutralization and hemagglutination-inhibition antibodies following influenza vaccination of HIV-infected and HIV-uninfected pregnant women

**DOI:** 10.1371/journal.pone.0210124

**Published:** 2018-12-31

**Authors:** Marta C. Nunes, Adriana Weinberg, Clare L. Cutland, Stephanie Jones, David Wang, Bonnie Dighero-Kemp, Min Z. Levine, Niteen Wairagkar, Shabir A. Madhi

**Affiliations:** 1 Department of Science and Technology/National Research Foundation, Vaccine Preventable Diseases and Medical Research Council, Respiratory and Meningeal Pathogens Research Unit, Johannesburg, South Africa; 2 Faculty of Health Sciences, University of the Witwatersrand, Johannesburg, South Africa; 3 Department of Pediatrics, Medicine and Pathology, University of Colorado, Aurora, Colorado, United States of America; 4 Influenza division, centre for Diseases Control and Prevention, Atlanta, Georgia, United States of America; 5 The Bill & Melinda Gates Foundation, Seattle, Washington, United States of America; Public Health England, UNITED KINGDOM

## Abstract

**Background:**

We previously reported that despite HIV-infected pregnant women had modest humoral immune responses to inactivated influenza vaccine (IIV) measured by hemagglutination-inhibition (HAI) assay, the observed vaccine efficacy against influenza disease was higher than predicted by HAI; suggesting that IIV may confer protection to HIV-infected individuals by additional mechanisms. We evaluated the response to IIV by microneutralization (MN) and HAI assays and correlated both methods in HIV-infected and HIV-uninfected pregnant women.

**Methods:**

MN and HAI antibodies were measured pre-vaccination and approximately one-month post-vaccination in 80 HIV-infected and 75 HIV-uninfected women who received IIV. Geometric mean titers (GMTs), fold-change in titers and seroconversion rates were determined for the three influenza stains in the vaccine.

**Results:**

After vaccination there were significant increases in MN and HAI GMTs for the three vaccine strains in both HIV-infected and HIV-uninfected women. HIV-infected women had, however, a lower immune response compared to HIV-uninfected. Fold-increases were 2 to 3-times higher for MN assay compared to HAI assay for the influenza-A strains. Also a higher percentage of women seroconverted by MN than by HAI assay for the influenza-A strains. There was high positive correlation between MN and HAI assays, except for the B/Victoria strain at pre-vaccination.

**Conclusions:**

In general, the MN assay was more sensitive than the HAI assay. Microneutralization antibodies might correlate better with protection against influenza infection.

## Introduction

Annual influenza vaccination is recommended for groups at high-risk for severe influenza infections, including pregnant women and HIV-infected individuals [1]. In a placebo-randomized clinical trial we reported that immunization of HIV-uninfected and HIV-infected pregnant women with seasonal trivalent inactivated influenza vaccine (IIV) was safe, immunogenic and partially protected the vaccinated women against polymerase chain reaction (PCR)-confirmed influenza-illness [2]. Although influenza vaccination during pregnancy increases maternal hemagglutination-inhibition (HAI) antibodies, we reported that HIV-infected pregnant women had inferior humoral HAI response compared to HIV-uninfected women, including lower percentages with HAI titers ≥1:40 post-vaccination (49%-67% vs. 85%-98%, respectively) [3]. The lower HAI response in HIV-infected women did not, however, translate into inferior vaccine efficacy against PCR-confirmed influenza compared to HIV-uninfected women (57.7% vs. 50.4%, respectively) [2, 3]. These data indicate that IIV may confer protection to HIV-infected individuals by mechanisms other than HAI antibodies.

The HAI assay is the most commonly used methodology to determine responses following influenza vaccination because of its relative correlation with protection, as well as its ease of performance, good standardization between laboratories and low price [4]. This assay detects antibodies to the viral surface protein hemagglutinin (HA) that can prevent agglutination to sialic-acid residues on erythrocytes, HAI titers only measure antibodies that block receptor binding of the virus to host cells, and it is only a correlate of the capacity of antibodies to inhibit viral infection of host cells in the respiratory tract [5]. Another serological assay for determining influenza-specific antibodies is microneutralization (MN); this functional assay directly measures antibodies that neutralize influenza virus infection, by evaluating the ability of antibodies to prevent virus entry, and viral replication that can occur in infection-permissive mammalian cells lines *in vitro*.[6]. The MN assay therefore measures the functional capability of antibodies at a specific dilution, rather than just the total quantity. Compared to HAI, MN assay measures a broader repertoire of antibodies [7]. Furthermore, MN assays have been shown to detect strain-specific antibodies against the immunodominant HA head domain and antibodies targeting the more conserved HA stalk domain. HA stalk-specific antibodies are known to mediate a number of important effector functions through their Fc-region including antibody-dependent cellular cytotoxicity (ADCC) and antibody-dependent phagocytosis (ADP) [8]. Assays measuring neutralizing antibodies reportedly are also more sensitive than HAI assays for detection of low level of antibodies and for diagnosing influenza infection [9–11]. The MN assay has, however, higher technical complexity, is more difficult to perform for clinical laboratories, and standardization across laboratories can be problematic.

Despite the extensive use of these two laboratory methods, only a few studies have formally compared immune responses to inactivated vaccine by both assays [10, 12–14], including in HIV-infected individuals [15–17].

The aim of this analysis was to measure and compare neutralizing and HAI antibody responses following influenza vaccination in HIV-infected and HIV-uninfected pregnant women enrolled into an IIV trial in 2011; and evaluate the correlation between the two serological assays.

## Materials and methods

### Influenza vaccine cohort

The two randomized, double-blind, placebo-controlled trials of IIV in HIV-infected and HIV-uninfected pregnant women have been described [2]. Briefly, pregnant women in their second/third trimester with documented HIV-1 infection status were randomized (1:1) to receive IIV or placebo in two parallel cohort studies. Maternal blood was collected in the HIV-infected women and in a sub-set of HIV-uninfected participants immediately prior to and at approximately one month after vaccination, then again at delivery, and at 24 weeks post-delivery. Enrolment occurred between 3^rd^ March and 2^nd^ June 2011. Active surveillance for respiratory illness and PCR-confirmed influenza-illness was performed from the time of enrolment up to 24 weeks post-delivery. The influenza vaccine used in the study was the recommended by WHO for the southern hemisphere in 2011 (A/California/7/2009 [A/H1N1pdm09], A/Victoria/210/2009 [A/H3N2], B/Brisbane/60/2008-like virus [B/Victoria lineage]; Vaxigripe; Sanofi-Pasteur, Lyon, France).

Both studies were approved by the Human Research Ethics Committee of the University of the Witwatersrand (101106 and 101107) and conducted in accordance with Good Clinical Practice guidelines, participants provided written informed consent. The studies were registered at ClinicalTrial.gov (NCT01306682 and NCT01306669).

### Neutralization and hemagglutination-inhibition assays

Whole blood samples from study participants were collected in heparin tubes (BD Vacutainer). Pilot studies were performed to verify that serum and plasma specimens collected in lithium heparin tubes yielded similar results by both HAI and MN assays prior to testing the plasma samples collected during this study. In a previous report we described the study participants’ immune responses to IIV and antibody kinetics measured by HAI assays that were performed at the University of Colorado (Aurora, Colorado, USA) [3]. Here we used archived plasma samples collected prior to vaccination (pre-IIV) and one month following IIV administration (post-IIV) to assess immune responses to IIV using MN assay. Only IIV-recipients with samples available at both time-points were included in the current analysis. The MN assays were performed in the Influenza Division research laboratory at Centers for Disease Control and Prevention (Atlanta, Georgia, USA). For MN assays [18], plasma samples were first heat inactivated, and then serial 2-fold dilutions were made starting at an initial 1:10 dilution. Influenza viruses (100 50% tissue culture infective doses, TCID50) were added to the plasma dilutions, incubated at 37°C with 5% CO_2_ for 1 hour, and used to infect 1.5×10^4^ Madin-Darby canine kidney cells per well. After overnight incubation, viral infection was quantified by an enzyme-linked immunosorbent assay (ELISA), using monoclonal antibodies specific to the influenza viruses’ nucleoproteins (NP); for influenza clone A1 and A3 blend (Millipore) were used and for influenza B clone B2 and B4 blend, (Millipore). Neutralizing antibody titers were defined as the reciprocal of the highest dilution of plasma that yielded at least 50% neutralization; reported titers are geometric mean titers (GMTs) from at least 2 replicates.

Immunogenicity assessments included: GMTs of HAI and MN antibodies pre-IIV and post-IIV for each of the three influenza stains in the vaccine, fold-increase in titers from pre-IIV to post-IIV, subjects with titers ≥1:40 pre-IIV and post-IIV, participants who seroconverted defined by ≥4-fold titer increase from pre-IIV to post-IIV with post-IIV titers ≥1:40.

### Statistical analysis

Demographic categorical variables were compared by Chi-square test, continuous normal distributed variables by Student’s t-test and non-normal distributed variables by Mann-Whitney test. Geometric mean titers, fold-change in titers and the corresponding 95% confidence interval (95%CI) were estimated using logarithmic transformation and compared between the study cohorts by multivariate linear regression. The percentages of women with titers ≥1:40 or who seroconverted were compared between the study cohorts by multivariate logistic regression. Participants who had a PCR-confirmed influenza episode between the two immunogenicity visits were excluded from the analyses of the putative strain for post-IIV measures. The frequency of participants with different antibody titers was examined by reverse cumulative distribution plots. Correlations between HAI and MN titers at both study visits were determined on log-transformed data by Spearman’s rank correlation. P-values <0.05 were considered statistically significant. Analyses were performed using STATA version 13.1 (College Station, TX, USA).

## Results

### Study cohorts

Eighty HIV-infected and 75 HIV-uninfected women who were vaccinated during pregnancy had pre-IIV and post-IIV paired plasma samples available for MN assays. No differences between the two cohorts were noted in baseline characteristics, except that a lower percentage of HIV-infected women (17.5% vs. 32.0%; p = 0.036) were primigravida and HIV-infected women were vaccinated slightly later in pregnancy (27.7 weeks of gestation vs. 26.3 weeks of gestation; p = 0.038) (Table 1). The mean time between the two immunogenicity visits was 32.3 days for HIV-infected women and 33.0 days for HIV-uninfected women. HIV-infected women had a median CD4+-cell count of 412cells/mm^3^ (interquartile range [IQR]: 274, 572), median HIV viral load of 1679 copies/ml (IQR: 90, 16619), 19.3% had undetectable HIV viral load and 80% were on antiretroviral therapy at the time of vaccination (Table 1).

**Table 1:** Demographic characteristics of HIV-infected and HIV-uninfected women at vaccination.

	HIV-infected N = 80	HIV-uninfected N = 75	p-value
Mean age (SD); years	27.2 (4.9)	26.3 (5.2)	0.241^b^
Median BMI (IQR)	28 (26, 33) [65]	29 (26, 32) [46]	0.94 ^c^
Mean Gestational Age (SD); weeks	27.7 (3.9)	26.3 (4.6)	0.038^b^
Median gravidity (IQR)	2 (2, 3)	2 (1, 2)	0.027^c^
Primigravida; n (%)	14 (17.5)	24 (32.0)	0.036^d^
Median CD4+ cell count (IQR); cells/mm^3^	412 (274, 572) [79]	-	-
Women with CD4+ cell count <200 cells/mm^3^; n (%)	8 (10.1) [79]	-	-
Women with CD4+ cell count 200–350 cells/mm^3^; n (%)	24 (30.4) [79]	-	-
Women with CD4+ cell count 350–500 cells/mm^3^; n (%)	24 (30.4) [79]	-	-
Women with CD4+ cell count >500 cells/mm^3^; n (%)	23 (29.1) [79]	-	-
Median HIV-1 viral load (IQR); copies/ml	1679 (90, 16619) [78]	-	-
Women with HIV-1 viral load ≤40 copies/ml; n (%)	15 (19.2) [78]	-	-
Women on antiretroviral therapy^a^; n (%)	64 (80.0)	-	-
Mean days between vaccination and 1 month post-vaccination visit (SD)	32.3 (7.2)	33.0 (6.8)	0.544^b^

IQR: interquartile range; SD: standard deviation.

^a^Includes participants on prevention of mother-to-child HIV transmission specific antiretroviral therapy and participants on highly active antiretroviral treatment numbers in brackets are the number of participants with available information. P-values calculated by:

^b^Student’s t-test,

^c^Mann-Whitney test or

^d^Chi-square test.

### Neutralization and HAI antibody levels in the two study cohorts

Three HIV-infected women had a PCR-confirmed A/H1N1 infection at 5, 6 and 14 days after vaccination; these participants were excluded from the post-IIV A/H1N1 analyses.

**Fig 1:**
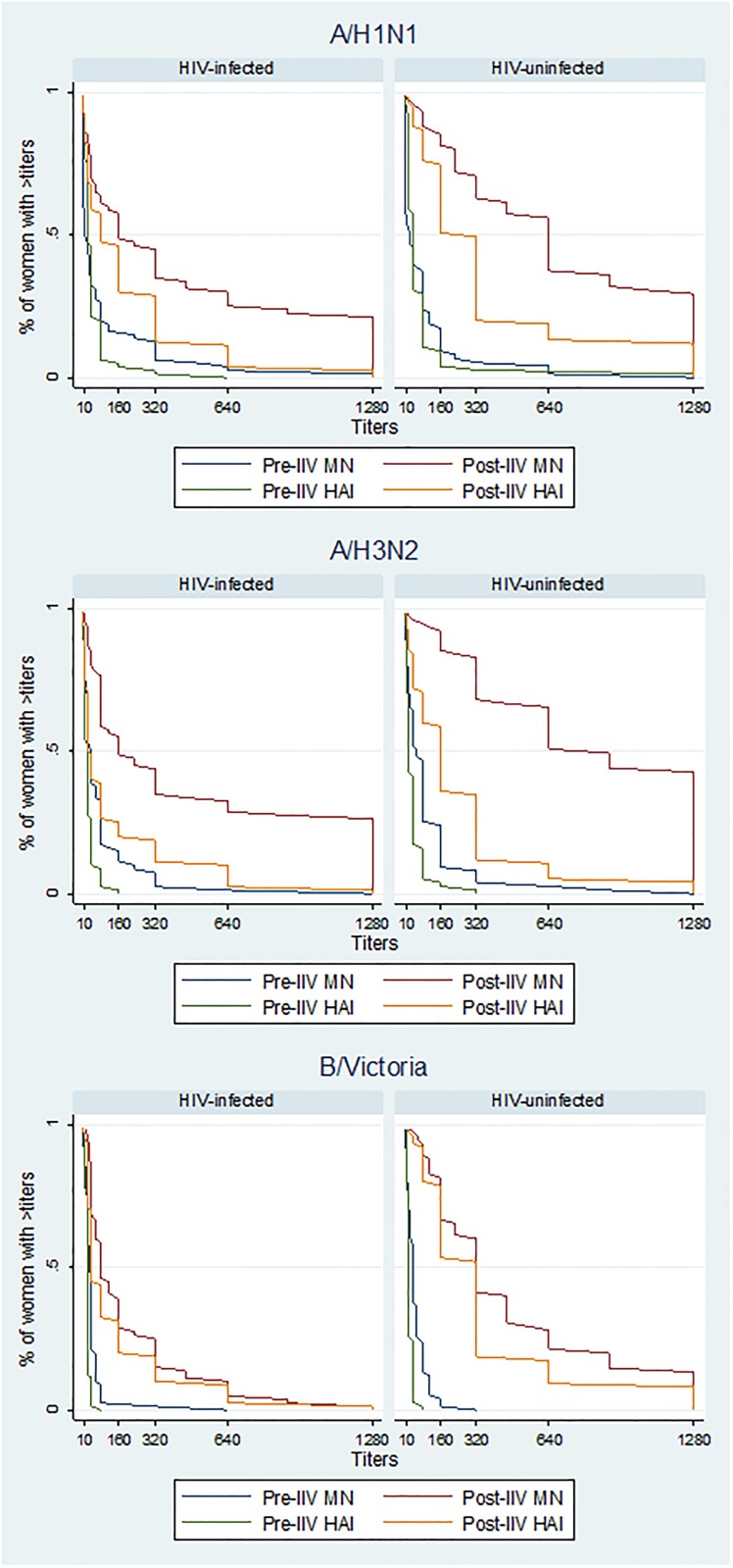
Reverse cumulative distribution curves of neutralization and hemagglutination-inhibition titers for the three influenza vaccine strains at the two study visits.

At baseline HIV-infected women compared to HIV-uninfected women had lower MN and HAI titers [3], and similarly a lower percentage of participants had titers ≥1:40; although only for A/H3N2 HAI antibodies these comparisons reached significance (p = 0.007) (Table 2 and Fig 1).

**Table 2:** Serological measurements assessed by microneutralization and hemagglutination-inhibition assays in HIV-infected and HIV-uninfected women who received influenza vaccine while pregnant.

	A/H1N1		A/H3N2		B/Victoria	
	HAI	MN	HAI	MN	HAI	MN
**GMTs pre-IIV (95%CI)**						
**HIV-infected**	28.0 (22.5, 35.0)	22.9 (15.8, 33.2)	17.7** (14.7, 21.4)	34.8 (26.0, 46.5)	18.5 (16.7, 20.5)	27.7 (22.8, 33.7)
**HIV-uninfected**	39.6 (31.5, 49.9)	26.2 (18.0, 38.0)	25.2 (20.4, 31.1)	49.7 (36.9, 66.7)	20.8 (18.4, 23.4)	35.6 (28.8, 44.0)
**GMTs post-IIV (95%CI)**						
**HIV-infected**	73.1* (51.5, 103.9)	142.6* (94.1, 216.0)	44.8* (31.9, 62.9)	193.2* (137.1, 272.2)	66.7* (50.2, 88.7)	106.9* (83.2, 137.4)
**HIV-uninfected**	215.1 (167.0, 277.0)	447.2 (344.9, 580.0)	124.7 (93.1, 166.9)	583.5 (464.0, 733.7)	229.4 (184.7, 285.0)	316.8 (254.2, 394.7)
**Fold-change in GMTs**						
**HIV-infected**	2.6* (2.0, 3.3)	6.0* (4.0, 8.9)	2.5* (2.0, 3.3)	5.6* (4.1, 7.6)	3.6* (2.7, 4.7)	3.9* (2.9, 5.1)
**HIV-uninfected**	5.4 (4.1, 7.1)	17.1 (12.0, 24.4)	4.9 (3.7, 6.6)	11.8 (9.2, 15.0)	11.1 (8.7, 14.0)	8.9 (6.8, 11.6)
**Women with titers ≥1:40 pre-IIV; n (%), (95%CI)**						
**HIV-infected**	38 (47.5) (36.2 59.0)	29 (36.3) (25.8, 47.8)	22 (27.5)*** (18.1, 38.6)	41 (51.3) (39.8, 62.6)	10 (12.5) (6.2, 21.8)	39 (48.8) (37.4, 60.2)
**HIV-uninfected**	44 (58.7) (46.7, 70.0)	35 (46.7) (35.1, 58.6)	32 (42.7) (31.3, 54.6)	49 (65.3) (53.5, 76.0)	19 (25.3) (16.0, 36.7)	44 (58.7) (46.7, 69.9)
**Women with titers ≥1:40 post-IIV; n (%), (95%CI)**						
**HIV-infected**	52 (67.5)* (55.9, 77.8)	60 (77.9)** (67.0, 86.6)	40 (50.0)* (38.6, 61.4)	69 (86.3)*** (76.7, 92.9)	57 (71.3)* (60.0, 80.8)	70 (87.5)*** (78.2, 93.9)
**HIV-uninfected**	72 (96.0) (88.8, 99.2)	73 (97.3) (90.7, 99.7)	64 (85.3) (75.3, 92.4)	73 (97.3) (90.7, 99.7)	73 (97.3) (90.7, 99.7)	74 (98.7) (92.8, 100)
**Women who seroconverted**^a^; **n (%), (95%CI)**						
**HIV-infected**	30 (39.0)* (28.0, 50.8)	49 (63.6)*** (51.9, 74.3)	30 (37.5)** (26.9, 49.0)	45 (56.3)* (44.7, 67.3)	35 (43.8)* (32.7, 81.7)	36 (45.0)* (33.8, 56.5)
**HIV-uninfected**	50 (66.7) (54.8, 77.1)	60 (80.0) (69.2, 88.4)	45 (60.0) (48.0, 71.1)	65 (86.7) (76.8, 93.4)	68 (90.7) (81.7, 96.2)	55 (73.3) (61.9, 82.9)

MN: microneutralization; HAI: hemagglutination-inhibition; CI: confidence intervals GMTs: Geometric mean titers; IIV: inactivated influenza vaccine.

^a^Seroconversion defined as ≥4-fold titer increase from pre-IIV to post-IIV with post-IIV titers ≥1:40.

Significant differences in responses between HIV-infected and HIV-uninfected women are indicated:

*adjusted p-value ≤0.001,

**adjusted p-value ≤0.01,

***adjusted p-value <0.05. All p-values adjusted for gravidity and gestational age at vaccination; p-values for the post-IIV, fold-change and seroconversion calculations were also adjusted for baseline titers.

After vaccination there were significant increases in MN and HAI titers for the three vaccine strains in both study cohorts. Fold-increases were 2 to 3-times higher for MN titers compared to HAI except for B/Victoria strain. Similarly a higher percentage of women had MN titers ≥1:40 compared to HAI titers ≥1:40; and a higher percentage of women displayed seroconversion assessed by MN than by HAI assay for A/H1N1 and A/H3N2, although the opposite was seen for B/Victoria among HIV-uninfected women (Table 2). The greater sensitivity of the MN assay to measure response to vaccination was particular heightened in the HIV-infected cohort where an extra 24% and 19% of participants demonstrated seroconversion for A/H1N1 and A/H3N2, respectively, compared to seroconversion assessed by HAI assay (63.6% vs. 39.0% for A/H1N1 and 56.3% vs. 37.5% for A/H3N2, respectively). In the HIV-uninfected cohort an extra 13% and 26% of participants demonstrated seroconversion for A/H1N1 and A/H3N2, respectively by MN assay (80.0% vs. 66.7% for A/H1N1 and 86.7% vs. 60.0% for A/H3N2, respectively). In both unadjusted analyses and adjusting for gravidity, gestational age at vaccination and baseline titers, immune responses to vaccination were significantly lower in the HIV-infected cohort than in HIV-uninfected women regarding GMTs post-IIV, fold-change in titers from pre-IIV to post-IIV, the percentage of women with titers ≥1:40 or the percentage of women who seroconverted for both neutralization and HAI antibodies (Table 2).

Fig 1 displays the frequency of participants pre-IIV and post-IIV with different antibody titers assessed by both methods for the three vaccines strains, elucidating that in general a higher percentage of women had higher MN titers compared to HAI titers.

**Fig 2:**
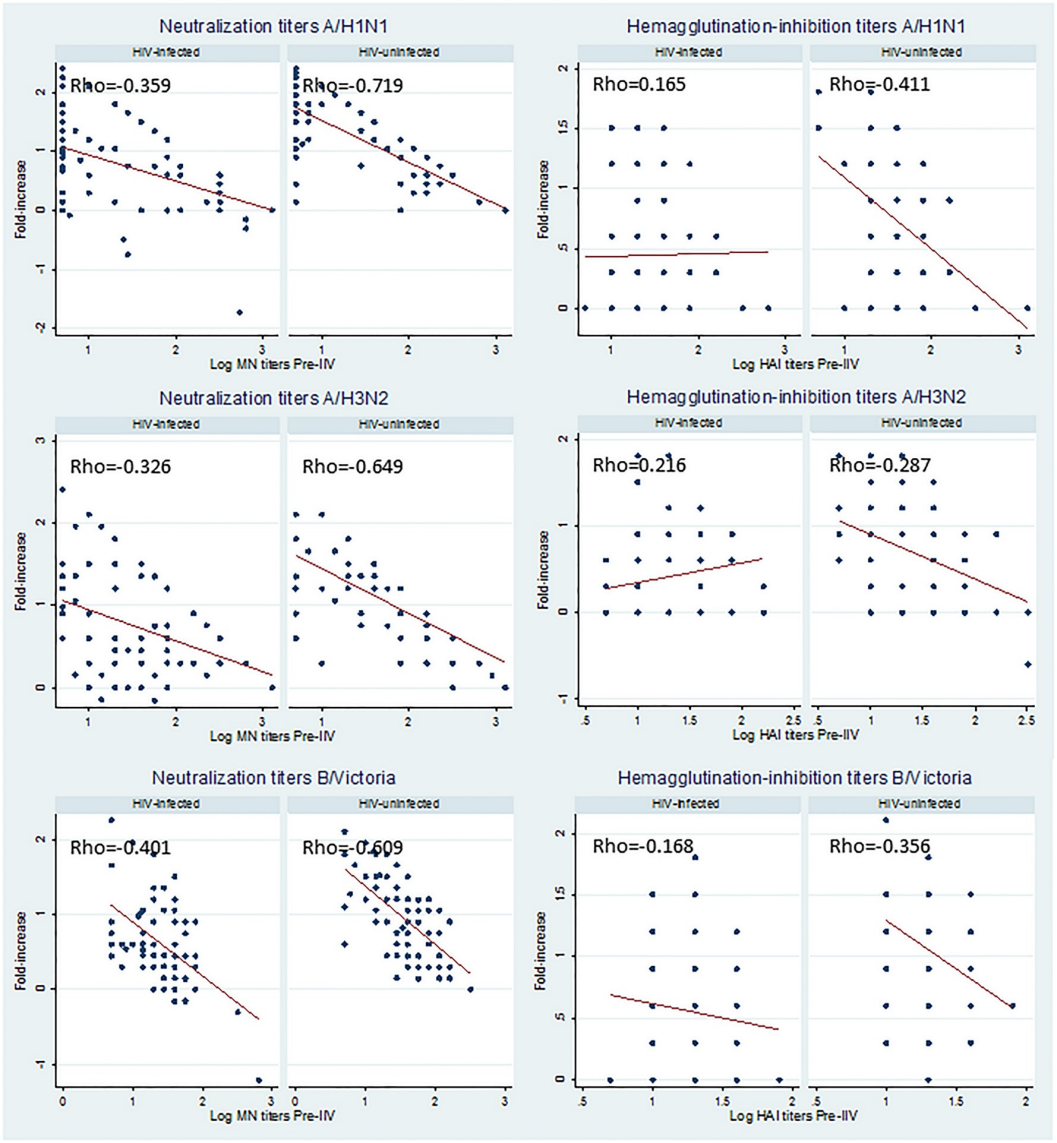
Correlation between fold-increase in neutralization or hemagglutination-inhibition titers post-vaccination and baseline titers for the three influenza vaccine strains. Footnote: for neutralization titers all p-values<0.01, for hemagglutination-inhibition titers all p-values≤0.01 for HIV-uninfected women p = 0.971 and all p-values>0.05 for HIV-infected women.

HIV-uninfected women with higher baseline immunity displayed a lower fold-increase in MN and HAI titers at the post-IIV visit for the three vaccine strains. These negative correlations were however stronger for the MN assay (Fig 2). In HIV-infected women higher pre-IIV titers were also associated with a reduced fold-increase in MN antibodies, but not in HAI antibodies (Fig 2).

**Fig 3:**
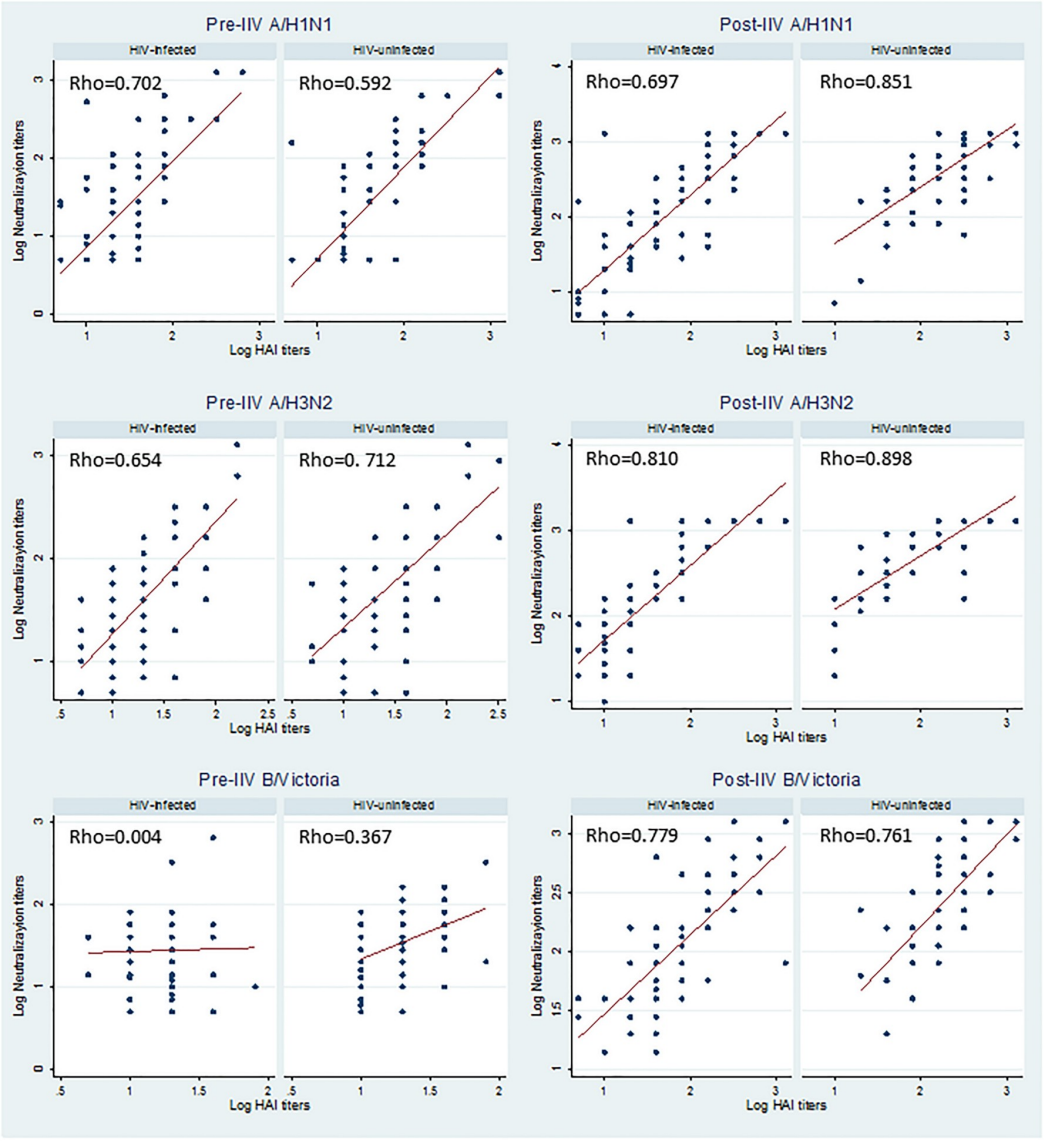
Correlation between log transformed neutralization and hemagglutination-inhibition titers for the three influenza vaccine strains at the two study visits. Footnote: all p-values<0.001, except for B/Victoria pre-IIV in HIV-infected women p = 0.971 and HIV-uninfected women p = 0.0012.

Correlation analyses between MN and HAI antibody levels showed in general strong correlations, with reciprocal MN titers being slightly higher than the corresponding HAI titers. Positive correlations between the two assays were detected pre-IIV among HIV-uninfected women for A/H1N1 and A/H3N2 (Spearman’s rank correlation coefficient [rho] = 0.70 and 0.63, respectively; p<0.001 for both) but weaker for B/Victoria (rho = 0.37; p = 0.001). In HIV-infected women positive correlations were detected for A/H1N1 and A/H3N2 (rho = 0.59 and 0.67, respectively; p<0.001 for both) but there was no correlation between MN and HAI titers for B/Victoria (rho = 0.004; p = 0.97) pre-IIV (Fig 3). Correlations post-IIV were strong for the three vaccine strains in both HIV-uninfected (rho = 0.69–0.81; p<0.001 for the three strains) and HIV-infected women (rho = 0.76–0.90; p<0.001 for the three strains) with correlation coefficients higher than pre-IIV (Fig 3).

### Influenza infection in the two study cohorts

Three HIV-infected women had PCR-confirmed A/H1N1 infection 44–49 days after vaccination (14–29 days after post-IIV visit; Table 3, case numbers 4–6). At the post-IIV visit two of these participants (case numbers 4 and 6) had MN titers ≤1:28 and HAI titers of 1:20 for A/H1N1; the third woman had MN and HAI titers of 1:453 and 1:160, respectively. The three HIV-infected women with PCR-confirmed A/H1N1 infection before the post-IIV visit (case numbers 1–3) all had MN titers ≤1:28 at baseline, two had HAI titers ≤1:20, and one had an HAI titer of 1:40 (Table 3).

**Table 3:** Antibody titers in HIV-infected and HIV-uninfected women who developed PCR-confirmed influenza disease.

	Case number	Days after vaccination	Days between influenza episode and preceding blood draw	Influenza type	MN titer at visit preceding the influenza episode	HAI titer at visit preceding the influenza episode
HIV-infected	1	5	5	A/H1N1	1:5	1:10
	2	6	6	A/H1N1	1:28	1:40
	3	14	14	A/H1N1	1:5	1:20
	4	44	16	A/H1N1	1:28	1:20
	5	45	14	A/H1N1	1:453	1:160
	6	49	29	A/H1N1	1:24	1:20
	7	141	106	A/H3N2	1:1280	1:320
HIV-uninfected	8	83	53	A/H3N2	1:20	1:10

MN: microneutralization; HAI: hemagglutination-inhibition.

One HIV-uninfected woman had a PCR-confirmed A/H3N2 infection 83 days following vaccination (53 days after post-IIV visit). At the post-IIV visit this participant had MN titers of 1:10 and HAI titers of 1:20 for A/H3N2 (Table 3).

## Discussion

We show that the MN assay was in general more sensitive than the HAI assay in detecting influenza-specific antibody seroconversion post-vaccination, including in HIV-infected individuals. Overall, there was strong correlation between MN and HAI results. Other studies have also demonstrated close association between the two assays [11, 14, 17], although the relationship between HAI titers and corresponding MN titers may be different for different influenza strains. The use of MN assay in clinical trials of vaccines is uncommon but its enhanced sensitivity has been demonstrated before [9–11, 14, 16]; although a recent phase I randomized clinical trial in Serbia assessing sero-responses to seasonal IIV found that GMTs and fold-increase in titers post-vaccination were higher by HAI than MN assay [19]. The controversial results with HAI in the literature may be due to the high technical variability of the assay added to the biological variability of the host. The technical variability of the HAI assay is linked to the source of the antigen, of the red blood cells used for the hemagglutination and the operator subjectivity. Quality control programs for standardization of influenza HAI or MN assays are not currently available, but they would be extremely helpful in ensuring comparability of studies.

Traditionally, HAI antibody titers ≥1:40 are associated with 50% reduction in the risk of influenza infection or disease in healthy adults [20, 21]. Less is known about the MN titer threshold that correlates with protection, but it has been suggested that effectiveness estimates against PCR-confirmed influenza are higher with the MN assay at similar titer thresholds [14, 22]. A household study in Hong Kong, assessing the ability of MN and HAI antibodies to predict protection against PCR-confirmed A/H3N2 infection in children and adults reported that titers of 1:40 by MN assay correlated with 49% protection while by HAI were associated with only 31% [22]. Moreover a recent study found that HAI titers of 1:40 for A/H1N1 and A/H3N2 in children corresponded to MN titres of approximately 1:200 and 1:140, respectively [14]. The sample size of our study was too small to determine what MN-titer had a protective effect, but of the eight women with a PCR-confirmed influenza infection during the entire study period, six had MN titers ≤1:28 to the putative strain within 53 days of infection; and in only two HIV-infected women MN titers were 1:453 and 1:1280.

Similar to our results two studies assessing the immunogenicity of inactivated monovalent influenza A/H1N1 2009 pandemic vaccines in HIV-infected children and young adults (n = 39) or adults (n = 84) found that post-vaccination GMTs and seroconversion rates were higher for MN than HAI assay [15, 16]. Influenza infections in immunocompromised patients are associated with prolonged illness and viral shedding, and increased morbidity and mortality [23, 24]. The ability of HIV-infected individuals to mount a protective immunological response to influenza vaccine has been evaluated in a limited number of studies that provided evidence for the poor immunogenicity of influenza vaccines in this population [25–28]. As we described before for HAI antibodies [3], the immune response to IIV in HIV-infected women assessed by MN assay was significantly lower than that observed in HIV-uninfected women; the similar vaccine efficacy observed in the two study cohorts [2] could, however, be due to the fact that post-vaccination MN GMTs were >1:100 for the three strains and this may well have been above the protective threshold.

The ability of the MN assay to detect functional antibodies able to neutralize the virus may imply that this assay measures a greater repertoire of antibodies involved in protection [7]. It is also possible that other immune mechanisms, such as HA stalk-specific antibodies, NA inhibition antibodies, non-neutralizing but functional antibodies, and cell mediated immune responses, may have contributed to protection. Ideally other types of antibodies should have been also investigated such as antibodies with Fc-mediated effector functions (like ADCC and ADP), HA stalk antibodies and NA antibodies, since especially the latter have been correlated with reduction of influenza clinical outcome measures [29, 30].

In general, low baseline antibody levels have been associated with higher fold-increases after vaccination [31]. Especially in adults, when assessing humoral responses to influenza vaccines the influence of pre-vaccination titers either due to natural infection or previous vaccination should be taken into account when assessing response with MN or HAI assays.

A limitation of our study was that the infecting viruses were not sequenced; if the infecting strains were drifted compared to the vaccine strains, the HAI and MN titers against the vaccine strains are probably not very relevant for protection. The fact that the two serological assays were performed in different laboratories during different periods is a limitation of our study, although we obtained good correlations between the two methods.

The European medicines agency has recently issued new guidelines on licensing of novel influenza vaccines in Europe emphasizing the importance of quantifying not only HAI antibodies but also functional antibodies as a measure of vaccine immunogenicity. Furthermore, the guidelines state that the protective threshold of HAI titers ≥1:40 no longer should be used [32]. This is particular relevant for special populations such as the HIV-infected where mechanisms other than HAI antibodies may play a major role in protection.
